# Detection of Colistin Resistance in *Pseudomonas aeruginosa* Using the MALDIxin Test on the Routine MALDI Biotyper Sirius Mass Spectrometer

**DOI:** 10.3389/fmicb.2021.725383

**Published:** 2021-08-31

**Authors:** Katy Jeannot, Katheryn Hagart, Laurent Dortet, Markus Kostrzewa, Alain Filloux, Patrick Plesiat, Gerald Larrouy-Maumus

**Affiliations:** ^1^UMR 6249 Chrono-Environnement, UFR Sciences Médicales et Pharmaceutiques, University of Bourgogne-Franche Comté, Besançon, France; ^2^French National Reference Centre for Antibiotic Resistance, Besançon, France; ^3^Department of Bacteriology, University Hospital of Besançon, Besançon, France; ^4^Department of Life Sciences, Faculty of Natural Sciences, MRC Centre for Molecular Bacteriology and Infection, Imperial College London, London, United Kingdom; ^5^Department of Bacteriology-Hygiene, Bicêtre Hospital, Assistance Publique – Hôpitaux de Paris, Le Kremlin-Bicêtre, France; ^6^EA7361 “Structure, Dynamic, Function and Expression of Broad Spectrum β-lactamases,” LabEx Lermit, Faculty of Medecine, Paris-Sud University, Le Kremlin-Bicêtre, France; ^7^Bruker Daltonik GmbH, Bremen, Germany

**Keywords:** MALDI mass spectrometry, lipid A, colistin, *Pseudomonas aeruginosa*, clinical isolate

## Abstract

Colistin is frequently a last resort treatment for *Pseudomonas aeruginosa* infections caused by multidrug-resistant (MDR) and extensively drug resistant (XDR) strains, and detection of colistin resistance is essential for the management of infected patients. Therefore, we evaluated the recently developed MALDIxin test for the detection of colistin resistance in *P. aeruginosa* clinical strains using the routine matrix-assisted laser desorption ionization (MALDI) Biotyper Sirius system. The test is based on the detection by mass spectrometry of modified lipid A by the addition of 4-amino-l-arabinose (l-ara4N) molecules on one or two phosphate groups, in strains resistant to colistin. Overproduction of l-Ara4N molecules is mainly due to the constitutive activation of the histidine kinase (PmrB) or the response regulator (PmrA) following an amino-acid substitution in clinical strains. The performance of the test was determined on a panel of 14 colistin-susceptible and 14 colistin-resistant *P. aeruginosa* clinical strains, the reference strain PAO1 and positive control mutants PmrB (V28G), PmrB (D172), PhoQ (D240–247), and ParR (M59I). In comparison with the broth microdilution (BMD) method, all the susceptible strains (*n*=14) and 8/14 colistin-resistant strains were detected in less than 1h, directly on whole bacteria. The remaining resistant strains (*n*=6) were all detected after a short pre-exposure (4h) to colistin before sample preparation. Validation of the method on a larger panel of strains will be the next step before its use in diagnostics laboratories. Our data showed that the MALDIxin test offers rapid and efficient detection of colistin resistant *P. aeruginosa* and is thus a valuable diagnostics tool to control the spread of these emerging resistant strains.

## Introduction

*Pseudomonas aeruginosa* is an opportunistic pathogen well-known for infections associated with intensive care units. It is one of the most frequent cause of acute pulmonary healthcare-associated infections, and severe infections particularly in immunocompromised patients (Vincent et al., 2009). Its intrinsic resistance to many antibiotics combined with its facility to accumulate a diversity of resistance mechanisms increasingly limits therapeutic options (Horcajada et al., 2019). Thus, polymyxins (polymyxin B or colistin) are used as a last resort for the treatment of *P. aeruginosa* infections caused by multidrug-resistant (MDR) and extensively drug resistant (XDR) strains (Sader et al., 2017; Doi, 2019; Walters et al., 2019; Ahmadian et al., 2020; Mirzaei et al., 2020). Unfortunately, resistance to colistin has emerged. In *P. aeruginosa*, acquired resistance to colistin mostly results from the addition of one or two 4-amino-l-arabinose (l-Ara4N) molecules to the 1 and/or 4' phosphate groups on the lipid A, the anchor of the LPS in the outer membrane (Bhat et al., 1990; Fernandez et al., 2013). While the *P. aeruginosa* genome contains an *eptA* like gene, the addition of phosphoethanolamine to lipid A or the LPS core has not been reported, unlike in *Enterobacterales* and *Acinetobacter baumannii* (Nowicki et al., 2015). The synthesis and the transport of l-Ara4N molecules is encoded by the large *arnBCADTEF-UgD* operon (simplified as *arn*), which is dependent on a complex regulatory network comprising at least 5 two-component systems (PmrA/PmrB, PhoP/PhoQ, ParR/ParS, CprR/CprS, and ColR/ColS; Fernandez et al., 2010, 2012; Needham and Trent, 2013; Nowicki et al., 2015). Furthermore, mutations in chromosomal genes encoding histidine kinase or response regulators of these two-component systems result in constitutive activation of the *arn* operon. However, in *P. aeruginosa* clinical strains, the genetic events most associated with colistin resistance are amino-acid substitutions leading to a gain of function of the PmrB protein (Barrow and Kwon, 2009; Schurek et al., 2009; Bolard et al., 2019). Although, the *mcr* genes have been widely reported in *Enterobacterales*, they have not currently been identified in *P. aeruginosa* strains, except in the chromosome of one clinical isolate strain (*mcr-5*; Snesrud et al., 2018).

Although, strains of *P. aeruginosa* resistant to polymyxins are still rare, their detection is one of the key issues to improve the treatment of patient infected with MDR and XDR *P. aeruginosa* strains (Diekema et al., 2019). Unfortunately, the methods currently available in routine laboratories for the detection of resistance to colistin in *P. aeruginosa*, still rely on bacterial growth in the presence of polymyxins. These procedures require 16–20h in culture, whether determining susceptibility to colistin minimal inhibitory concentration (MIC) using the broth microdilution method (BMD; reference method), or the colistin broth disk elution and colistin agar test methods recently accepted by the CLSI (2020). Only two test have reported the detection of colistin resistance strains in less than 3h (Sadek et al., 2020; Sorensen et al., 2020). The first is a biochemical test (Rapid Polymyxin/*Pseudomonas* NP test) based on the change in color of the bromocresol (yellow to purple/violet) following the production of basic metabolites during the growth of the strain in the presence of colistin (Sadek et al., 2020), and a fast lipid analysis technique (FLAT) directly on a matrix-assisted laser desorption ionization (MALDI) plate. However, both approaches have issues: possible misinterpretation of the colorimetric test, and potential cross contamination with the on-target hydrolysis in the FLAT method.

Therefore, there is an urgent need to develop a fast and robust assay to detect colistin-resistant *P. aeruginosa* strains. Recently, we developed a rapid technique using matrix-assisted laser desorption ionization time-of-flight (MALDI-TOF) to rapidly detect colistin resistance using whole cells, the MALDIxin test (Dortet et al., 2018a,b). The MALDIxin test has now been optimized for *Escherichia coli*, *Klebsiella pneumoniae*, *A. baumannii*, and *Salmonella enterica* (Dortet et al., 2018a,b, 2019, 2020; Furniss et al., 2019). The aim of this study is to evaluate the performance of the optimized MALDIxin test using a routine MALDI mass spectrometer (in comparison to the BMD method), to detect colistin-resistant *P. aeruginosa*.

## Materials and Methods

### Bacterial Strains

From the *Pseudomonas* collection of the French National Reference Centre for Antibiotic Resistance (Besançon, France), 14 colistin susceptible (MIC≤2mg/L) and 14 colistin resistant clinical strains (MIC>2mg/L) were selected. All the strains were genotypically-unrelated, and isolated from 25 health institutions distributed throughout France. In addition, two *pmrB* mutants (AB8.2, AB16.2), one *parR* mutant (PAOW2), and one *phoQ* mutant (AB8.4) derivate form the *P. aeruginosa* reference strain PAO1 were included as positive controls (Table 1; Muller et al., 2011; Bolard et al., 2019). The wild-type strain PAO1 was used as negative control.

**Table 1:** Characteristics and results of the MALDIxin test on *P. aeruginosa* strains.

Name of strain	MIC to colistin (mg/L)	Colistin resistance mechanism	PRR MALDIxin results	References
*Reference strains*				
PAO1	1	–	0.00±0.00/0.02±0.01^*^	Tenover, 2000
AB8.2	128	PmrB (V28G)	0.87±0.25	Bolard et al., 2019
AB16.2	128	PmrB (Δ172)	1.17±0.22/2.12±0.07^*^	Bolard et al., 2019
AB8.4	4	PhoQ (Δ240–247)	0.00±0.00/0.44±0.02^*^	This study
PAOW2	4	ParR (M59I)	0.29±0.05/0.28±0.01^*^	Muller et al., 2011
*Colistin susceptible clinical strains*				
185715	1	–	0.00±0.00/0.00±0.00^*^	This study
185716	1	–	0.00±0.00/0.00±0.00^*^	This study
218401	1	–	0.00±0.00/0.00±0.00^*^	This study
218418	1	–	0.00±0.00/0.00±0.00^*^	This study
218419	0.5	–	0.00±0.00/0.00±0.00^*^	This study
218420	1	–	0.00±0.00/0.00±0.00^*^	This study
218422	0.5	–	0.00±0.00/0.00±0.00^*^	This study
218423	0.5	–	0.00±0.00/0.00±0.00^*^	This study
218424	2	–	0.00±0.00/0.07±0.01^*^	This study
218427	1	–	0.00±0.00/0.00±0.00^*^	This study
218428	0.5	–	0.00±0.00/0.00±0.00^*^	This study
218429	0.5	–	0.00±0.00/0.00±0.00^*^	This study
218435	0.5	–	0.00±0.00/0.00±0.00^*^	This study
218437	1	–	0.00±0.00/0.03±0.01^*^	This study
*Colistin resistant clinical strains*				
142243	128	PmrB, (Q105P)^b^	0.00±0.00/0.22±0.01^*^	Bolard et al., 2019
152739	16	PmrB (V264A)^a^	0.39±0.04	Bolard et al., 2019
153038	64	PmrB (D47N)^b^	0.67±0.03	Bolard et al., 2019
153091	128	PmrA (L21I)	0.86±0.07	Bolard et al., 2019
163795	128	PmrB (G188D)^b^	0.23±0.01	Bolard et al., 2019
174536	4	PmrB (V136E)^b^	0.00±0.01/2.03±0.13^*^	Bolard et al., 2019
174660	64	PmrB (G121P, V313A)^b^	0.60±0.44	Bolard et al., 2019
174782	4	PmrB (H33Y)^a^	0.98±0.14	Bolard et al., 2019
175058	4	PmrB (D45N, G362S)^a^	0.24±0.01	Bolard et al., 2019
175101	32	PmrB (R92H, G123S)^b^	0.19±0.07	Bolard et al., 2019
185345	4	–	0.00±0.00/0.87±0.07^*^	This study
185374	4	–	0.00±0.00/0.22±0.01^*^	This study
185819	128	PhoQ (R275X)	0.22±0.06	This study
196337	4	–	0.00±0.00/0.34±0.03^*^	This study

a Mutation Y345H associated with polymorphism in the protein PmrB.

b Mutations S2P, A4T, V6A, V15I, G68S, and Y345H associated with polymorphism in the protein PmrB.

–, no mutation has been identified in genes *cprS*, *cprR*, *parS*, *parR*, *pmrA*, *pmrB*, *phoP*, *phoQ*, *colS*, and *colR*.

* PRR obtained after colistin induction of the strains.

### Susceptibility Testing

MICs were determined in triplicate by the BMD using colistin sulfate (Sigma Aldrich, Saint Quentin Fallavier, France) and cation-adjusted Mueller Hinton broth (MHB) from Becton Dickinson (Pont-de-Claix, France) according to the European Committee on Antimicrobial Susceptibility Testing (EUCAST) recommendations (EUCAST, 2019). Results were interpreted using EUCAST breakpoints (≤2mg/L; >2mg/L).

### Whole Genome Sequencing

Four clinical strains (185345, 185374, 185819, and 196337) for which the mutation responsible for colistin resistance had not been identified and characterized were genome sequenced. From an overnight bacterial culture, total DNA was extracted using the PureLink Genomic DNA Mini kit (ThermoFischer Scientific). The library preparation using NextEra® XT DNA preparation kit (Illumina, San Diego, CA, United States) and sequencing by Illumina NextSeq500 system (2×150-bp paired end reads) were performed by the “Plateforme de Microbiologie Mutualisée” (PibNet) at Institut Pasteur (Paris, France). Reads were assembled with Shovill-Spades v3.14.0. To identify mutations in genes associated with colistin resistance in *P. aeruginosa* (*cprR*, *cprS*, *colR*, *colS*, *parR*, *parS*, *phoP*, *phoQ*, *pmrA*, and *pmrB*), sequences from clinical strains were mapped to sequences of reference strains PAO1, and P, A14, and a large collection of 77 clinical strains susceptible to colistin using the NRC bioinformatic pipelines based on SNIPPY v4.6.0.

### Nucleotide Sequence Accession Number

The nucleotide sequences reported in this study and corresponding to the entire chromosome of strains 185345, 185374, 185819, and 196337 have been deposited in the GenBank nucleotide database under accession no JAGJAGMWR0000000000, JAGMWQ0000000000, JAGMWP0000000000, and JAGMWO0000000000, respectively.

### MALDIxin Test

*Pseudomonas aeruginosa* cells were exposed or not for 4-h to a subinhibitory (1/2 MIC) concentration of colistin. A 10 μl inoculation loop of bacteria, grown on Mueller-Hinton agar for 18–24h, was resuspended in 200μl of water. Mild-acid hydrolysis was performed on 100μl of this suspension, by adding 100μl of 2% *v*/*v* acetic acid and incubating the mixture at 98°C for 30min. Hydrolyzed cells were centrifuged at 17,000 *×g* for 2min, the supernatant was discarded, and the pellet was washed three times with 300μl of ultrapure water and resuspended to a density of McFarland 20 as measured using a McFarland Tube Densitometer. A volume of 0.4μl of this suspension was loaded onto the MALDI target plate and immediately overlaid with 1.2μl of Norharmane matrix (Sigma-Aldrich) solubilized in chloroform/methanol (90:10 *v*/*v*) to a final concentration of 10mg/ml. For external calibration, 0.5μl of calibration peptide was loaded along with 0.5μl of the given calibration matrix (peptide calibration standard II, Bruker Daltonik, Germany). The samples were loaded onto a disposable MSP 96 target polished steel BC (Bruker Part-No. 8280800).

The bacterial suspension and matrix were mixed directly on the target by pipetting then dried gently under a stream of air. The spectra were recorded in the linear negative-ion mode (laser intensity 95%, ion source 1=10.00kV, ion source 2=8.98kV, lens=3.00kV, detector voltage=2,652V, pulsed ion extraction=150ns). Each spectrum corresponded to ion accumulation of 5,000 laser shots randomly distributed on the spot. The spectra obtained were processed with default parameters using FlexAnalysis v.3.4 software (Bruker Daltonik, Germany).

### Data Analysis

The negative mass spectrum was scanned between *m*/*z* 1,300 and *m*/*z* 2,000 in the negative linear ion mode. Manual peak picking at masses relevant to colistin resistance was performed on the obtained mass spectra and the corresponding signal intensities at these defined masses was determined. The percentage of modified lipid A was calculated by dividing the sum of the intensities of the lipid A peaks attributed to addition of l-Ara4N (*m*/*z* 1,577.9, *m*/*z* 1,593.9, *m*/*z* 1,708.9, and *m*/*z* 1,724.9) by the intensity of the peaks corresponding to native lipid A (*m*/*z* 1,446.7 and *m*/*z* 1,462.7). All mass spectra were generated and analyzed in technical triplicate (i.e., measurements of each sample were repeated three times).

### Statistical Analysis

All experiments were carried out in biological duplicates. Data were compared two-by-two using unpaired Welch’s *t*-test. Values of *p*<0.05 were considered statistically different.

## Results and Discussion

**Figure 1:**
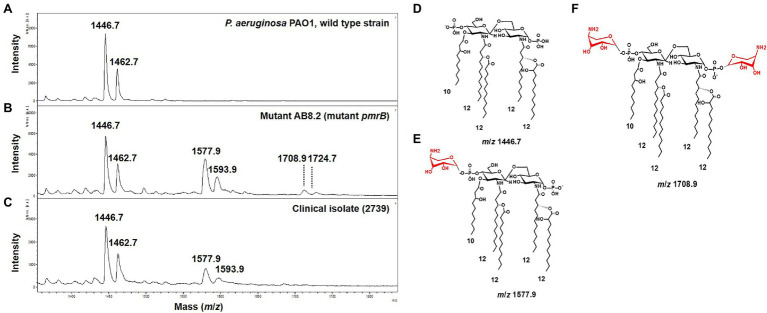
Representative mass spectra of susceptible and modified *Pseudomonas aeruginosa* lipid A acquired using the linear negative-ion mode of a matrix-assisted laser desorption ionization (MALDI) Biotyper Sirius system (Bruker Daltonics). **(A)** Susceptible *P. aeruginosa* PAO1 lipid A is detected as two major peaks at *m*/*z* 1,446.7 and *m*/*z* 1,462.7. **(B)** Lipid A from mutant *pmrB* (AB8.2) with additional peaks at *m*/*z* 1,577.9, *m*/*z* 1,593.9, *m*/*z* 1,708.9, and *m*/*z* 1,724.9 corresponding to 4-amino-L-arabinose (L-Ara4N) addition on the penta-acylated lipid A (peaks at *m/z m/z* 1,446.7 and *m*/*z* 1,462.7). **(C)** Lipid A from colistin-resistant *P. aeruginosa* clinical isolates is modified by L-Ara4N, which are detected as additional peaks at *m*/*z* 1,577.9, *m*/*z* 1,593.9. **(D)** Diagram of *P. aeruginosa* lipid A at *m*/*z* 1,446.7. **(E)** Diagram of *P. aeruginosa* lipid A at *m*/*z* 1,577.9. L-Ara4N residue is shown in red. **(F)** Diagram of *P. aeruginosa* lipid A at *m*/*z* 1,708.9. L-Ara4N residues are shown in red.

To assess the ability of the MALDIxin test to detect colistin-resistance in *P. aeruginosa* on a MALDI biotyper Sirius system, we tested a panel of 33 *P. aeruginosa* strains, including four isogenic *P. aeruginosa* PAO1 mutants representing the most frequently mutated genes involved in colistin resistance in this species. These included the reference strain PAO1, 14 colistin-resistant clinical strains (MIC from 4 to 128mg/L), and 14 colistin-susceptible clinical strains (MIC from 0.5 to 2mg/L; Table 1). The mass spectrum of colistin susceptible *P. aeruginosa* reference strain PAO1 is dominated by two peaks assigned to penta-acyl bis-phosphorylated lipid A (Figure 1A). The peak at *m*/*z* 1,446.7 is assigned to penta-acyl bis-phosphorylated lipid A, which corresponds to the presence of one 3-OH-C10:0 fatty acyl chain, three C12:0 fatty acyl chain and one 2-OH C12:0 fatty acyl chain. The lipid A structure at *m*/*z* 1,462.7 differs from that at *m*/*z* 1,446.7 only by the addition of one hydroxyl group at 3 position (3-OH) of one C12:0 fatty acyl chain. Both forms have frequently been reported in *P. aeruginosa* strains (Ernst et al., 2006; Moskowitz et al., 2012; Figure 1D). In comparison with the parental strain PAO1, four additional peaks at *m*/*z* 1,577.9, *m*/*z* 1,593.9, *m*/*z* 1,708.9, and *m*/*z* 1,724.9 were observed in the *pmrB* mutant (mutant AB8.2), which is resistant to colistin (Figure 1B). The signals at *m*/*z* 1,577.9, and *m*/*z* 1,593.9, and at *m*/*z* 1,708.9 and *m*/*z* 1,724.9 correspond to the addition of one or two l-Ara4N molecules to the 4′- or/and 1-phosphate groups of the penta-acylated form respectively, resulting in an increase of +131 *m*/*z* compared to the native lipid A peaks (Figures 1B,E,F). Since mutations in genes encoding the two-component systems PmrAB, ParRS, and PhoPQ lead to the addition of l-Ara4N molecules, we did not observe any difference in mass spectra between the *pmrB* (AB8.2 and AB16.2), and *parR* (PAOW2) mutants, compared to the *phoQ* mutant (AB8.4). A similar spectrum is also observed for clinical isolates (Figures 1C,F). Based on the MALDIxin profile, we attempted to determine the Polymyxin Resistance Ratio (PRR) of the sum of the intensities of the peaks associated with modified lipid A over the intensity of the peak of native lipid A allowing an accurate distinction between polymyxin-susceptible and polymyxin-resistant isolates. Despite several attempts, no signal corresponding to a modified lipid A was detected. Although, the MIC of colistin was similar in *pmrB* mutants (128mg/L), the intensity of peaks was clearly higher in *pmrB* mutant AB8.2 (PRR 87±25%) than AB16.2 (PRR 2±0%), indicating that the intensity of peaks is not correlated with resistance level to colistin. The same observation was previously reported for *E. coli*, *Salmonella*, *K. pneumoniae*, and *A. baumannii* (Dortet et al., 2018b, 2019, 2020; Furniss et al., 2019). As expected, the peaks observed in the 14 clinical strains susceptible to colistin did not differ from those obtained with the strain PAO1 (data not shown), and the percentage of modified lipid A was equal to zero (Table 1).

**Figure 2:**
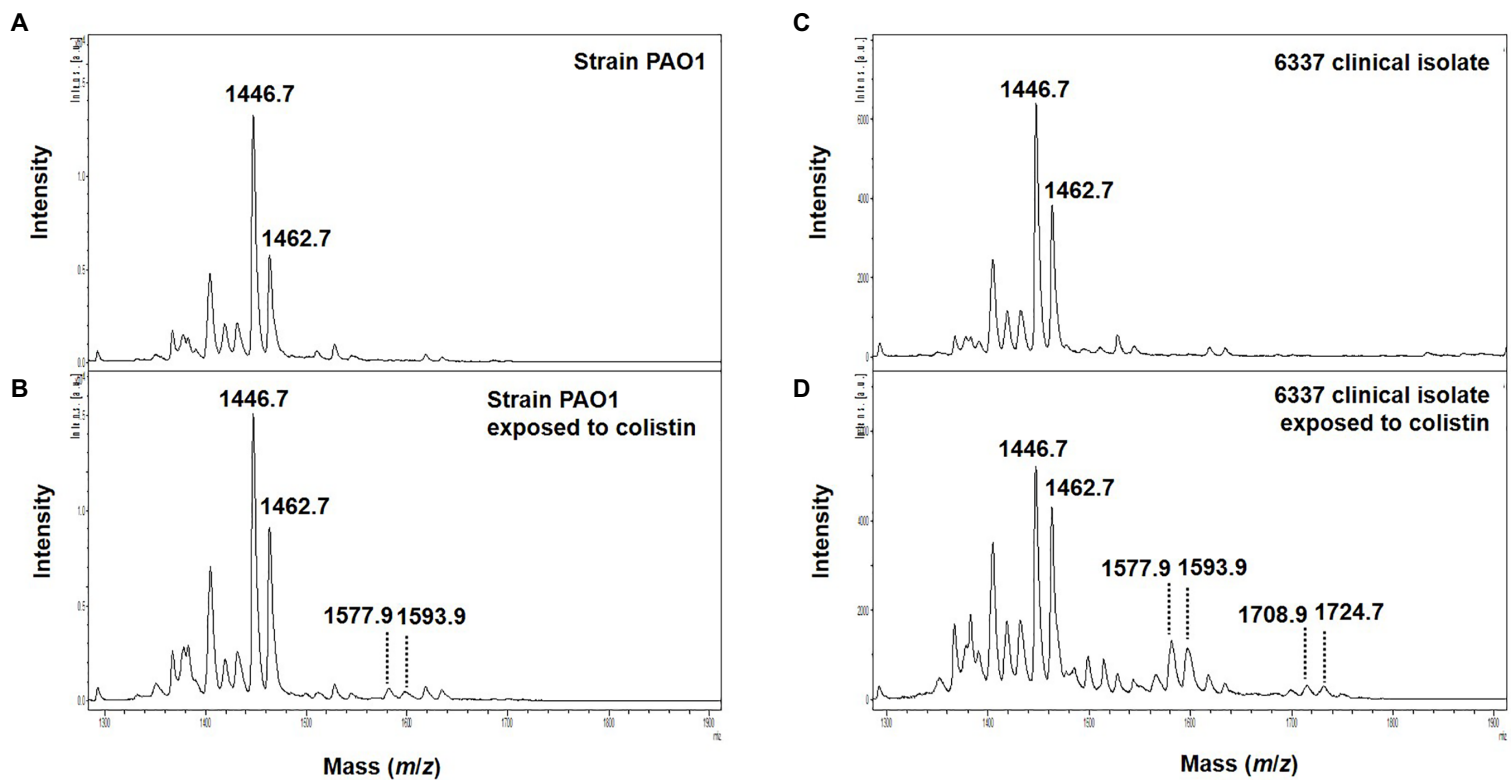
Representative mass spectra of colistin induced susceptible and modified *P. aeruginosa* lipid A acquired using the linear negative-ion mode of a MALDI Biotyper Sirius system (Bruker Daltonics). **(A)** Susceptible *P. aeruginosa* PAO1 lipid A is detected as two major peaks at *m*/*z* 1,446.7 and *m*/*z* 1,462.7. **(B)** Colistin induced susceptible *P. aeruginosa* PAO1 lipid A with additional peaks at *m*/*z* 1,577.9, *m*/*z* 1,593.9 corresponding to L-Ara4N addition on the penta-acylated lipid A (peaks at *m*/*z m*/*z* 1,446.7 and *m*/*z* 1,462.7). **(C)** Lipid A from colistin-resistant *P. aeruginosa* clinical isolate 6,337 is detected as two major peaks at *m/z* 1,446.7 and *m*/*z* 1,462.7. **(D)** Colistin induced colistin-resistant *P. aeruginosa* clinical isolate 6,337 lipid A with additional peaks at *m*/*z* 1,577.9, *m*/*z* 1,593.9, *m*/*z* 1,708.9, and *m*/*z* 1,724.9 corresponding to L-Ara4N addition on the penta-acylated lipid A (peaks at *m*/*z m*/*z* 1,446.7 and *m*/*z* 1,462.7).

**Figure 3:**
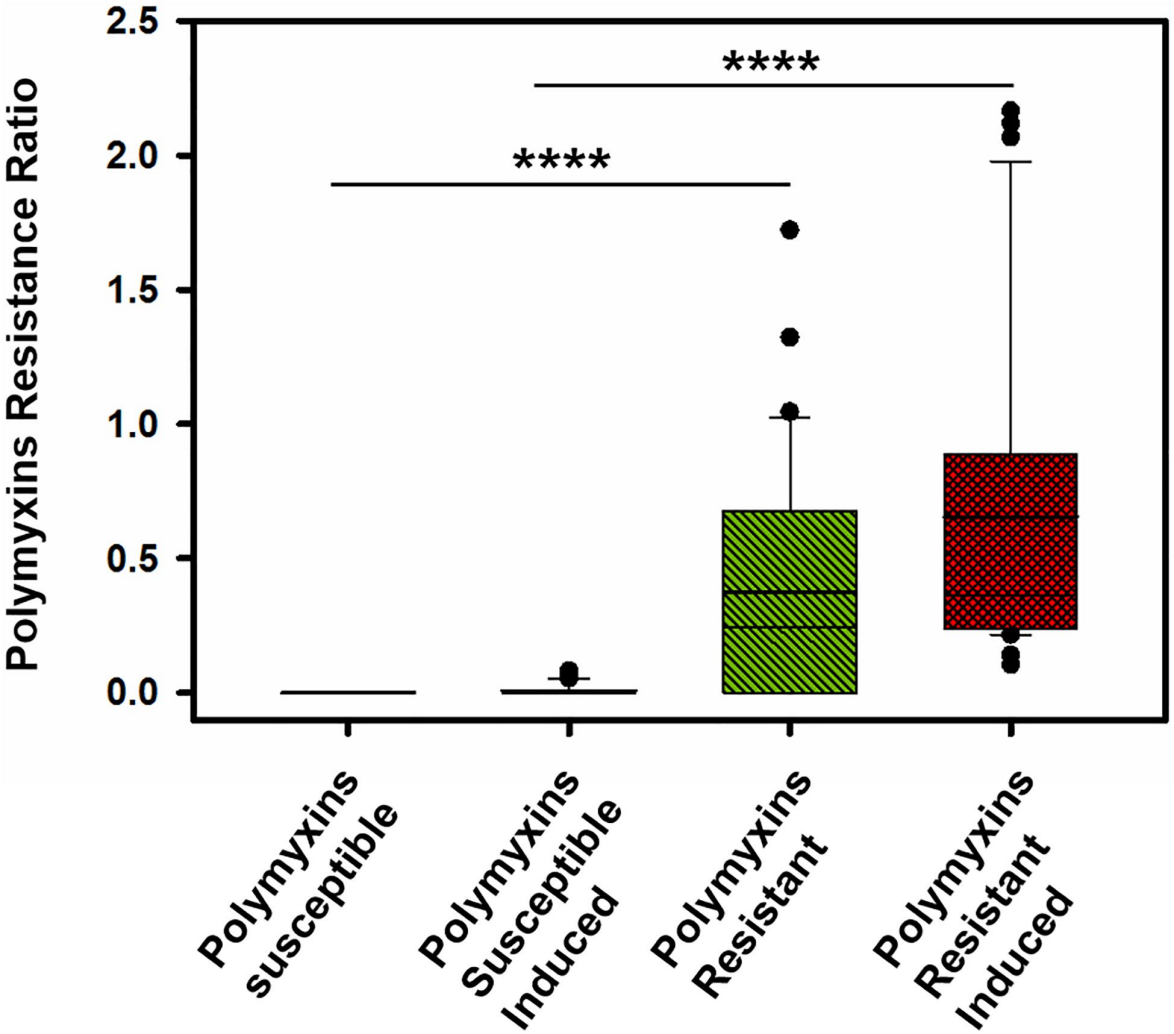
Distribution of the Polymyxin Resistance Ratios (PRRs) for the tested strains before and after induction with colistin for 4-h to a subinhibitory (1/2 MIC; ^****^p <0.0001).

Interestingly, in clinical strains resistant to colistin (*n*=14), the percentage of lipid A modified or PRR ranged from 0 to 90%. Although, five *P. aeruginosa* clinical strains and the *phoQ* mutant (mutant AB8.4) have a MIC higher than the breakpoint for colistin (>2μg/ml) including one strain with a MIC≥128mg/L (163795), we did not detect any modifications on the lipid A as reported by their null PRR (Table 1). Unlike for MIC determination, bacteria are not exposed to colistin when preparing bacteria for the MALDIxin test. It is likely that induction of the two-component systems PmrAB, CprRS, and ParRS is necessary to detect sufficient modification (beyond background) of lipid A in some strains (Moskowitz et al., 2004; Muller et al., 2011; Fernandez et al., 2012). Therefore, the strain PAO1, the *phoQ* mutant and the six strains were exposed to a sub-inhibitory concentration of colistin (½ MIC) for 4h, before the MALDIxin test and determination of the percentage of modified lipid A (Table 1). While the colistin susceptible reference strain exhibited 2% modified lipid A after colistin exposure, the *phoQ* mutant and colistin resistant clinical strains had more than 20% (Table 1; Figure 2). All the strains resistant to colistin were detected after a short exposure (4h) to colistin, confirming that for some strains, the sensitivity of the MALDIxin test can be enhanced by the induction of colistin resistance (Figure 3).

Here, we have demonstrated that resistance to colistin can be quickly and easily detected in clinical strains of *P. aeruginosa* using the MALDIxin assay. However, an adaptation of the protocol currently used will be necessary before its use in routine laboratories. Unlike other species such as *K. pneumoniae*, *A. baumannii*, *E. coli*, and *S. enterica*, the signal intensity corresponding to the modified lipid A can be masked in some strains resistant to colistin. The addition of colistin during sample preparation phase improves the detection of *P. aeruginosa* strains resistant to colistin. Despite this additional step in sample preparation, the technique remains rapid (less than 5h), comparing favorably to the BMD for determining the MIC of colistin. The MALDIxin method complements the panel of so-called rapid methods for detecting resistance to colistin in clinical strains of *P. aeruginosa*, including the Rapid Polymyxin/*Pseudomonas* NP test (Sadek et al., 2020). MALDIxin is cost effective since it can be coupled with bacterial identification using the norharmane matrix with the MALDI Biotyper Sirius. One of the limitations of the study resides in the low number of strains tested, and further validation with an expanded panel is required. However, the most frequent mechanism responsible for colistin resistance in *P. aeruginosa* clinical strains (*pmrB* mutants) are included in this study, which supports the use of MALDIxin as a diagnostic for hospitalized patients.

## Data Availability Statement

The datasets presented in this study can be found in online repositories. The names of the repository/repositories and accession number(s) can be found in the article/supplementary material.

## Author Contributions

GL-M, LD, and KJ conceived the study, participated in its design, and performed the experiments. KJ provided the clinical isolates. KJ, KH, LD, MK, AF, PP, and GL-M wrote the manuscript. All authors contributed to the article and approved the submitted version.

## Conflict of Interest

LD, AF, and GL-M are co-inventors of the MALDIxin test for which a patent has been filed by Imperial Innovations (WO2018158573). MK is employee of Bruker, the manufacturer of the MALDI-TOF MS used in this study.

The remaining authors declare that the research was conducted in the absence of any commercial or financial relationships that could be construed as a potential conflict of interest.

## Publisher’s Note

All claims expressed in this article are solely those of the authors and do not necessarily represent those of their affiliated organizations, or those of the publisher, the editors and the reviewers. Any product that may be evaluated in this article, or claim that may be made by its manufacturer, is not guaranteed or endorsed by the publisher.
